# Oligo-DNA Custom Macroarray for Monitoring Major Pathogenic and Non-Pathogenic Fungi and Bacteria in the Phyllosphere of Apple Trees

**DOI:** 10.1371/journal.pone.0034249

**Published:** 2012-03-30

**Authors:** Ying-Hong He, Sayaka Isono, Makoto Shibuya, Masaharu Tsuji, Charith-Raj Adkar Purushothama, Kazuaki Tanaka, Teruo Sano

**Affiliations:** Faculty of Agriculture and Life Science, Hirosaki University, Hirosaki, Japan; U. S. Salinity Lab, United States of America

## Abstract

**Background:**

To monitor the richness in microbial inhabitants in the phyllosphere of apple trees cultivated under various cultural and environmental conditions, we developed an oligo-DNA macroarray for major pathogenic and non-pathogenic fungi and bacteria inhabiting the phyllosphere of apple trees.

**Methods and Findings:**

First, we isolated culturable fungi and bacteria from apple orchards by an agar-plate culture method, and detected 32 fungal and 34 bacterial species. *Alternaria*, *Aureobasidium*, *Cladosporium*, *Rhodotorula*, *Cystofilobasidium*, and *Epicoccum* genera were predominant among the fungi, and *Bacillus*, *Pseudomonas*, *Sphingomonas*, *Methylobacterium*, and *Pantoea* genera were predominant among the bacteria. Based on the data, we selected 29 major non-pathogenic and 12 phytopathogenic fungi and bacteria as the targets of macroarray. Forty-one species-specific 40-base pair long oligo-DNA sequences were selected from the nucleotide sequences of rDNA-internal transcribed spacer region for fungi and 16S rDNA for bacteria. The oligo-DNAs were fixed on nylon membrane and hybridized with digoxigenin-labeled cRNA probes prepared for each species. All arrays except those for *Alternaria*, *Bacillus*, and their related species, were specifically hybridized. The array was sensitive enough to detect 10^3^ CFU for *Aureobasidium pullulans* and *Bacillus cereus*. Nucleotide sequencing of 100 each of independent fungal rDNA-ITS and bacterial 16S-rDNA sequences from apple tree was in agreement with the macroarray data obtained using the same sample. Finally, we analyzed the richness in the microbial inhabitants in the samples collected from apple trees in four orchards. Major apple pathogens that cause scab, Alternaria blotch, and Marssonina blotch were detected along with several non-phytopathogenic fungal and bacterial inhabitants.

**Conclusions:**

The macroarray technique presented here is a strong tool to monitor the major microbial species and the community structures in the phyllosphere of apple trees and identify key species antagonistic, supportive or co-operative to specific pathogens in the orchard managed under different environmental conditions.

## Introduction

The microbial flora of plants, including organisms on the plant exterior as well as those in the interior, plays an important role in shaping the microbial ecosystems in the phyllosphere [1]–[3]. Both species richness and environmental complexity increase ecosystem functioning, suggesting that detailed knowledge of how individual species interact with complex natural environments is necessary to make reliable predictions about how alterations in the biodiversity affect the functioning of the ecosystem [4]. On the other hand, microbes, especially epiphytic fungi and bacteria surviving on crop plants, have been influenced by environmental changes such as those in temperature, rain, soil, nutrients, and even by agricultural practices, including spraying of chemical pesticides and fertilizer input.

The routine method of spraying chemical pesticides currently employed in modern agriculture has been long believed to have a more or less negative impact on the microbial diversity in the phyllosphere. Actually, on the basis of the findings of a pesticide program on the non-target epiphytic microbial population of apple leaves, it was reported that populations of bacteria, filamentous fungi, yeast, and actinomycetes varied annually and were reduced 10- to 1,000-fold in 1976 and up to 50-fold in 1977 on pesticide-treated leaves [5]–[6]. A similar study on the agrichemical impact on the growth and survival of non-target microorganisms in the phyllosphere of apple trees revealed that repeated agrichemical applications reduced the *in planta* microbial population 10- to 10,000-fold, suggesting that agrichemicals could affect the non-target, culturable surface microorganisms [7]. A seasonal comparative study of the effect of organic and integrated production systems on the culturable fungi of stored Golden Delicious apples was conducted in Switzerland; the findings revealed that organically produced apples had significantly higher frequencies of filamentous fungi, abundance of total fungi, and higher taxon diversity than the apples produced by integrated systems [8].

Changes in the epiphytic microflora, in turn, are believed to influence the incidence of crop diseases through competitive and/or antagonistic interactions with invading pathogens and pests. Evaluation of the microbial difference in species level in plants is anticipated to become more important because of the promotion of sustainable agriculture and integrated pest management. The traditional methods such as agar-plate culture method are time-consuming, and at times, require additional labor and skills for DNA extraction, polymerase chain reaction (PCR) amplification, and sequencing. Furthermore, potential uncertainty may persist in the differentiation and identification of colonies based on their macroscopic appearance. We also need to consider that not all the microbial inhabitants are culturable; moreover, the growth of some culturable organisms could be underestimated, because of their decreased activity in the medium and culture conditions being used.

To overcome these difficulties, we can utilize the findings from several studies that identified specific pathogens or multiple species by using DNA-based high-throughput techniques to establish risk assessment models or to monitor microbial diversity *in planta* under various environmental conditions; for example, identification and differentiation of the bacterial pathogens of potatoes [9], quantitative assessment of the growth of phytopathogenic fungi on various substrates [10], risk assessment of grapevine powdery mildew [11], diversity pattern of maize leaf epiphytic bacteria in relation to the plants that are genetically resistant to fungal pathogens [12], diagnostic DNA microarray for rapid identification of quarantine bacteria [13], characterization of complex communities of fungi and fungal-like protists [14], proteogenomic analysis of the physiology of phyllosphere bacteria [15], microarray screening of the variability of 16S–23S rRNA internal transcribed spacer region (ITS) in *Pseudomonas syringae* [16], and analysis of molecular battles between the plant and the pathogenic bacteria in the phyllosphere [17].

Here we first present the development of an oligo-DNA custom macroarray technique to detect and monitor the major pathogenic and non-pathogenic fungi and bacteria inhabiting the phyllosphere of apple trees, and then the application of the macroarray to analyze the richness of microbial inhabitants in orchards managed with different disease control measures such as intensive calendar spraying of chemical pesticides, reduced spraying of chemical pesticides, or natural farming practices.

## Materials and Methods

### Ethics statement

No specific permits were required for the described field studies. No specific permissions were required for these locations/activities, because of the owner's personal kind considerations on our researches. The field studies did not involve endangered or protected species.

### Apple orchards used for the analysis

Culturable fungi and bacteria inhabiting the phyllosphere (leaf surface and inner tissue spaces) of *Fuji* apple (*Malus×domestica*) trees were isolated by using an agar-plate culture method 16 times from May 2006 to October 2008 for samples from four apple orchards in Hirosaki City, Aomori Prefecture, Japan. The trees in Orchard A-chemical (intensive spraying of chemical pesticides; owned by Hirosaki University) were managed under normal cultivation conditions wherein chemical pesticides and fungicides were to be sprayed 11 times in 2009 growing season, according to calendar-based pest management (http://www.applenet.jp/). Chemicals sprayed include fenbuconazole, Score MZ (difenoconazole and mancozeb), ziram, thiram, iminoctadine triacetate, Aliette-C WP (captan), Flint Flowable25 (trifloxystrobin), cyprodinil, Antracol WG (propineb), calcium carbonate, and copper organic compounds as fungicide, and machine oil, organophosphorus compounds (e.g. chlorpyrifos), pyrethroid (e.g. cypermethrin), and neonicotinoid (e.g. clothianidin) as insecticide. In comparison, the trees in Orchard A-organic (Japan Agricultural Standard (JAS) organic; owned by Hirosaki University) in the neighboring field were grown under organic farming conditions for the past several years; *i.e.*, vinegar and acid water were sprayed periodically as alternatives to chemical pesticides. The trees in Orchard B-semi-chemical (reduced spraying of chemicals; owned by Makoto Takeya) were managed under special cultivation (*Tokubetsu Saibai* in Japanese) conditions in which the routine number of chemical pesticide sprays and amount of chemical fertilizer (nitrogen-based) were reduced by half. Chemicals sprayed include iminoctadine triacetate, Score MZ (difenoconazole and mancozeb), copper organic compounds, Stroby (kresoxim-methyl), and captan as fungicide, and machine oil, BT (*Bacillus thuringiensis*) spore, Confuser R (the mating disruptant), organophosphorus compounds (e.g. phenthoate), and neonicotinoid (e.g. acetamiprid) as insecticide. The trees in Orchard B-natural (natural farming; owned by Akinori Kimura) were managed under natural farming conditions without spraying any chemical fertilizers or pesticides, but were sprayed with specially formulated vinegar sprayings for the past 30 years. No specific permissions were required for these locations/activities, because of the owner's personal kind considerations on our researches.

### Isolation of the culturable fungi and bacteria inhabiting the phyllosphere of apple trees

Apple leaves (*ca.* 0.5 g) were immersed in 35 mL of distilled water and shaken vigorously for 1 min. The liquid was collected and centrifuged at 4,000×*g* for 15 min at room temperature to precipitate the fungi and bacteria, which were then dissolved in 5 mL of distilled water. Aliquots (200-µL each) were spread on multiple 9-cm Petridishes containing *ca.* 30 mL of potato dextrose agar (PDA) and King's B medium and incubated at 20°C and 25°C for the isolation of fungal and bacterial colonies, respectively. The fungal and bacterial colonies thus obtained were further purified by single-colony isolation method. The apple leaves were washed, as described above, sterilized for 1 min by immersion in 70% alcohol, air dried, and homogenized in 1.5 mL of distilled water. Aliquots (200-µL each) were spread, as described above, for isolating the fungi and bacteria present inside the leaves.

### Extraction, polymerase chain reaction, sequencing, and identification of fungi and bacteria

After several passages of single-colony isolation, the purified fungal and bacterial isolates were used for DNA isolation by using ISOPLANT II DNA extraction kit (Nippon Gene, Osaka, Japan), according to the manufacturer's instructions. An aliquot of DNA (100 ng in 2 µL) was used for PCR amplification of fungal rDNA-ITS with the primer set ITS1 (5′-TCCGTAGGTGAACCTGCGG-3′) and ITS4 (5′- TCCTCCGCTTATTGATATGC-3′) [18], and of bacterial 16S rDNA region with the primer set Bac16S-27F (5′-AGAGTTTGATCCTGGCTCAG-3′) and Bac16S-1525R (5′-AAAGGAGGTGATCCAGCC-3′). The amplified DNA was sequenced at Macrogen (Seoul, Korea), and the genus or species was determined by performing BLAST analysis of the sequence at the DNA Data Bank of Japan; those showing the sequence homology higher than 98% were identified as species.

### Direct nucleotide sequencing analysis of microbial rDNA population in the phyllosphere without the culture step

Apple leaves used for the analysis were the same to those described in details in the latter section on “Sampling of apple leaves and preparation of macroarray probe — in the phyllosphere of the apple orchards”. The two methods were used for the direct extraction of microbial DNAs from apple leaves.

#### Method 1

Apple leaves (0.5 g) were homogenized in 3 volumes (1.5 mL) of distilled water; centrifuged 3 times in a microcentrifuge (MX-150; Tomy Seiko Co., Ltd., Tokyo, Japan), the first at 1,000×*g* for 2 min, the second at 1,500×*g* for 2 min, and the third at 2,000×*g* for 2 min; and left undisturbed for 30 min. The supernatant was then collected and centrifuged at 15,000×*g* for 20 min to precipitate microbes inhabiting the phyllosphere of the apple trees. The precipitate was used for DNA extraction by using ISOPLANT II DNA extraction kit, according to the manufacturer's instructions. The extracted DNA was dissolved in 50 µL of distilled water.

#### Method 2

The preparation by Method 1 includes a large amount of chloroplast and mitochondorial DNAs of host origin. These DNAs could disturb especially bacterial 16S rDNA amplification, because they are also prokaryotic origin. To eliminate the chloroplast and/or mitochondrial from the preparation, apple leaves (0.5 g) were homogenized and fractioned according to Ikeda et al. [19]. Briefly, leaves were homogenized in BCP buffer (5 ml for 1 g leaf), centrifuged at 500×*g* for 1 minute, collected supernatant, centrifuged at 5,000×*g* for 1 minute, and collected the precipitates. The precipitate was dissolved in 1 ml BCP buffer, vigorously shaken for a second, and re-precipitated by centrifugation at 5,000×*g* for 1 minute. BCP buffer (1 ml) treatment was repeated, and the final precipitate was used for DNA extraction by ISOPLANT II.

The rDNA-ITS region for fungi was amplified by PCR using the primer set ITS1 and ITS4, and a part [ca. 500 base pair (bp)] of the 16S-rDNA for bacteria was amplified by the primer set Bac-27F and Bac-519R [20]. The amplified DNA fragments were ligated in pT7blue–T vector (Novagen, Merck KGaA, Darmstadt, Germany) for transformation of *Escherichia coli* (DH5α strain), and the transformant colonies obtained were used for the preparation of recombinant plasmid DNA for sequencing as above.

### Macroarray hybridization

#### Array sequences

On the basis of the nucleotide sequences obtained, 40-bp oligo-DNA sequences specific for each microorganism was selected from fungal rDNA-ITS and bacterial 16S-rDNA sequences as oligo-DNA arrays.

#### Macroarray preparation

The oligo-DNA arrays specially prepared in this experiment (FASMAC Co., Ltd., Kanagawa, Japan) were dissolved at a concentration of 1 µg/µL in a solution containing 50% (v/v) formamide (Wako), 35% (v/v) formaldehyde (Wako), and 1×saline-sodium citrate (SSC) buffer; denatured at 65°C for 15 min; diluted with 4 volumes of 20× SSC buffer; and then, aliquots (1-µL each) were spotted on a positively charged nylon membrane (Biodyne Plus, Pall Corporation, Mexico). Arrays were fixed on the membranes by ultraviolet (UV) cross-linking (120,000 µJ/cm^2^).

#### Preparation of digoxigenin (DIG)-labeled RNA probe for macroarray analysis

A combine of apple leaves (0.5 g) was treated as above in the Method 1 and 2, and a mixture DNA from various microbial inhabitants in the phyllosphere was prepared by using ISOPLANT II DNA extraction kit and dissolved in 50 µL of distilled water. Similarly, the total DNA of each fungal and bacterial isolate for the preliminary analysis was extracted from the cultured preparation.

The microbial DNAs were amplified by PCR in a 25-µL mixture containing 2 µL of total DNA extract, 2.5 µL of each dNTPs at 2.5 mM, 2.5 µL of 10× LA PCR buffer, 2.5 µL of 25 mM MgCl_2_, 0.25 µL of LA-Taq DNA polymarase (Takara Bio, Shiga, Japan), and 1 µL of each of the PCR primers (each 20 µM) Bac16S-27F and Bac16S-1525R (5′-AGAG-*TAATACGACTCACTATAGGG*-AAAGGAGGTGATCCAGCC-3′) for amplification of bacterial 16S rDNAs, and ITS1 and ITS4-T7 (5′-AGAG-*TAATACGACTCACTATAGGG*-TCCTCCGCTTATTGATATGC-3′) for fungal rDNA-ITS region. Bac16S-1525R and ITS4-T7 primers contained the promoter sequence for T7 RNA polymerase at the 5′-end (italicized letters).

Cycle parameters for PCR amplification were heat-denaturation at 94°C for 4 min, followed by 35 cycles of amplification (94°C for 1 min; 55°C, 1 min; and 72°C, 1 min), and a final extension at 72°C for 7 min. The amplified cDNAs were extracted twice by equal volumes of phenol∶chloroform (1∶1), precipitated by ethanol, and dissolved in 50 µL of distilled water.

A DIG-labeled cRNA probe was prepared in a 5.5-µL transcription mixture containing 2.5 µL of the PCR product (*ca.* 100 ng/µL), 0.5 µL of RNA-labeling mixture (Roche Diagnostics Japan, Tokyo, Japan), 1 µL of 5× T7 buffer (Invitrogen, Life Technologies Japan, Tokyo, Japan), 0.25 µL of 0.1 M dithiothreitol (DTT), 0.125 µL of RNase inhibitor (Wako), and 0.25 µL of T7 RNA polymerase (Invitrogen) by incubating at 37°C for 2 h. The transcription reaction was stopped by adding 0.5 µL of 0.2 M ethylenediaminetetraacetic acid (EDTA) (pH 8.0), 0.625 µL of 4 M LiCl, and 18.75 µL of 99.5% ethanol, and was then stored overnight at −30°C. DIG-labeled cRNAs were collected by centrifugation at 13,000×*g* for 10 min, washed in 70% ethanol, air dried, and dissolved in 25 µL of distilled water containing 0.05 µL of RNase inhibitor.

#### Hybridization

Array membrane (10×10 cm) was placed in a glass hybridization bottle and prehybridized in 5-mL hybridization buffer containing 5× SSC buffer, 1% Denhardt solution, 1% sodium dodecyl sulphate (SDS), and 25 mg of yeast tRNA (Roche Diagnostics) at 58°C for 1.5 h. DIG-labeled cRNA probes were heat-denatured at 95°C for 10 min, and an aliquot (5-µL) was then added to the hybridization solution. Hybridization was carried out at 58°C for at least 18 h. Membranes were washed twice for 15 min each in 70 mL (5 M NaCl, 0.8 M NaH_2_PO_4_, 0.1 M EDTA) at room temperature, and again twice for 15 min each in 70 mL of 1.5×SSPE (0.5% SDS) buffer at 58°C for 15 min. Membranes were then placed in 5-mL blocking solution containing 1% blocking reagent (Roche Diagnostics) in 0.1 M maleic acid (0.15 M NaCl, pH 7.5), incubated at room temperature for 30 min, and further incubated for 30 min after adding 2 µL of anti-DIG-alkaline phosphatase (AP) (Fab fragment) (Roche Diagnostics). Membranes were washed twice for 15 min each in 70 mL of washing buffer (0.1 M maleic acid (0.15 M NaCl, pH 7.5)) containing 0.3% Tween 20 (v/v) and immersed for 5 min in a 50-mL solution containing 0.1 M Tris-HCl (0.1 M NaCl and 0.05 M MgCl_2_, pH 9.5). Membranes were incubated with CSPD star (ready-to-use) (Roche Diagnostics) for 30 min–2 h in ChemiDoc XRS (Bio-Rad Laboratories Japan, Tokyo, Japan) to detect chemiluminescent signals.

#### Quantification

The signal intensity was quantified by Quantity One (Bio-Rad).

### Sampling of apple leaves and preparation of macroarray probe for the analysis of the seasonal changes in richness in the major microbial inhabitants in the phyllosphere of the apple orchards

Apple leaf samples were collected from the four orchards (A-chemical, A-organic, B-semi-chemical, and B-natural), which were the same orchards sampled for agar-plate culture experiments. Apple leaves were collected from these orchards at 2-week intervals from May 29 to October 29, 2009, i.e., 3 leaves from 1 position, 3 positions in 1 tree, and 3 trees in 1 orchard; namely, a total of 27 leaves per orchard. The 27 leaves were crushed into small pieces in the liquid nitrogen, mixed well, and an aliquot (ca. 0.5-g) was used for the harvest of microbial inhabitants in the phyllosphere (Method 1 and 2), followed by extraction of microbial DNAs (ISOPLANT II). Mixtures of fungal rDNA-ITS regions and/or bacterial 16S-rDNAs of the isolates obtained from the phyllosphere of the apple trees were amplified simultaneously or separately by PCR with primer sets specific to the fungal and bacterial species, and DIG-labeled RNA probes were finally transcribed.

## Results

### Detection and identification of culturable fungi and bacteria in the phyllosphere of apple trees by agar-plate culture method

A total of more than 150 each of independent culturable fungal and bacterial isolates were examined for sequencing, and 112 fungal and 135 bacterial informative sequences were obtained. All the fungal and bacterial species which showed the most high sequence similarity to those isolated from the phyllosphere of apple trees are listed in Table 1 and 2. They are identified at the genus or species level on the basis of the rDNA-ITS nucleotide sequence (*ca.* 500 bp) for fungi and 16S-rDNA sequence (*ca.* 1400 bp) for bacteria. A total of 32 different species (or unique sequences) in 31 fungal genera and 34 species in 22 bacterial genera were identified. The genera *Alternaria*, *Aureobasidium*, *Cladosporium*, *Rhodotorula*, *Cystofilobasidium*, and *Eoicoccum* in fungi and *Bacillus*, *Pseudomonas*, *Sphingomonas*, *Methylobacterium*, and *Pantoea* in bacteria were predominant.

**Table 1:** List of fungi species detected from four apple orchards by agar-plate culturing method in 2006–2008 seasons.

genus	species	identity (%)	accession No. matched	frequency	accession No. deposited
*Alternaria*	*alternata*	523/523 (100)	JF835810	12	AB693900
*Arthrinium*	*sacchari*	527/528 (99)	HQ914941	6	AB693901
*Aureobasidium*	*pullulans*	554/554 (100)	HQ909089	16	AB693902
*Biscogniauxia*	*latirim*	417/448 (93)	EF026135	1	AB693903
*Botryosphaeria*	*dothidea*	539/539 (100)	HQ730969	1	AB693904
*Botrytis*	*elliptica*	486/486 (100)	FJ169671	2	AB693905
*Cladosporium*	*tenuissimum*	491/492 (99)	JN689952	16	AB693906
*Coprinus*	*xanthothrix*	631/634 (99)	FJ755223	1	AB693907
*Curvularia*	*trifolii*	552/562 (98)	AF455446	1	AB693908
*Cryptococcus*	*victoriae*	452/453 (99)	AF444645	4	AB693909
*Cystofilobasidium*	*macerans*	547/555 (98)	AF444317	5	AB693910
*Epicoccum*	*nigrum*	483/484 (99)	DQ981396	5	AB693911
*Fusarium*	*chlamydosporum*	495/495 (100)	FJ426391	3	AB693912
*Gibberella*	*avenacea*	509/510 (99)	FJ224099	1	AB693913
*Hormonema*	*prunorum*	535/536 (99)	AJ244248	1	AB693931
*Leptosphaeria*	sp.	476/480 (99)	FN394721	1	AB693914
*Leptosphaerulina*	*australis*	490/490 (100)	JN712494	2	AB693915
*Microdiplodia*	sp.	561/564 (99)	EF432267	1	AB693916
*Monilinia*	sp.	427/430 (99)	AY805571	1	AB693917
*Mucor*	*racemosus*	564/580 (97)	AJ271061	1	AB693918
*Myrothecium*	*verrucaria*	538/541 (99)	EF211127	1	AB693919
*Nigrospora*	sp.	455/462 (98)	AM262341	2	AB693920
*Paraconiothyrium*	*variabile*	377/403 (93)	HM150642	4	AB693921
*Penicillium*	*mali*	526/528 (99)	AF527056	4	AB693922
*Phomopsis*	sp.	520/524 (99)	AB302248	3	AB693923
*Ramularia*	*pratensis*	466/478 (97)	EU019284	1	AB693924
*Rhodotorula*	*glutinis*	492/504 (98)	AY188373	6	AB693925
*Rhodotorula*	*laryngis*	504/510 (98)	AF444617	6	AB693926
*Sclerotinia*	*sclerotiorum*	541/541 (100)	AF455526	1	AB693927
*Stemphylium*	*solani*	500/504 (99)	EF104156	1	AB693928
*Xylaria*	sp.	523/527 (99)	AB255244	1	AB693930

They are identified at the genus or species level on the basis of the rDNA-ITS nucleotide sequence (*ca.* 500 bp) for fungi. “Identity” was shown by the number of nucleotide matched per number of nucleotide compared. “Frequency” indicates the numbers of detection out of 16 trials.

**Table 2:** List of bacteria species detected from four apple orchards by agar-plate culturing method in 2006–2008 seasons.

genus	species	identity (%)	accession No. matched	frequency	accession No. deposited
*Achromobacter*	*xylosoxidans*	1394/1413 (99)	AF511516	2	AB695331
*Acinetobacter*	sp.	1400/1423 (98)	JN887918	1	AB697151
*Agrobacterium*	*tumefaciens*	1373/1378 (99)	AB681363	1	AB695332
*Arthrobacter*	sp.	1407/1418 (99)	DQ519082	1	AB695333
	*cereus or thuringiensis*	1437/1438 (99)	JN315893	16	AB697152
*Bacillus*	*megaterium*	1414/1419 (99)	HQ202555	16	AB697153
*Bacillus*	*pseudomycoides*	1442/1457 (98)	AB681414	5	AB695334
*Bacillus*	*pumilus*	1443/1446 (99)	GU125624	8	AB695335
*Bacillus*	*subtilis*	1442/1450 (99)	HQ711983	7	AB697154
*Bradyrhizobium*	*elkanii*	1360/1364 (99)	AB672634	3	AB695336
*Burkholderia*	*fungorum*	1422/1437 (98)	FJ708122	3	AB695337
*Curtobacterium*	*flaccumfaciens*	1399/1419 (98)	AM410688	1	AB695338
*Dermacoccus*	sp.	1378/1386 (99)	JF905611	1	AB697155
*Gluconobacter*	*oxydans*	1364/1373 (99)	AB178421	1	AB695339
*Methylobacterium*	*suomiense*	1359/1378 (98)	AB175645	1	AB697157
*Methylobacterium*	*radiotolerans*	1342/1364 (98)	AY616142	4	AB697158
*Microbacterium*	*foliorum*	1414/1422 (99)	EU714371	2	AB695340
*Micrococcus*	*luteus*	1395/1403 (99)	HM755622	2	AB697159
*Paenibacillus*	*amylolyticus*	1377/1394 (98)	DQ313379	2	AB695341
*Paenibacillus*	*pasadenensis*	1395/1427 (97)	AB681404	2	AB697160
*Pantoea*	*agglomerans*	1348/1376 (97)	FJ357813	7	AB695342
*Pseudomonas*	*graminis*	1418/1426 (99)	Y11150	8	AB695343
*Pseudomonas*	*fluorescens*	1386/1392 (99)	JN679853	4	AB695344
*Pseudomonas*	*oryzihabitans*	1380/1401 (98)	AB681726	1	AB697161
*Pseudomonas*	*putida*	1397/1406 (99)	EU275363	7	AB697162
*Pseudomonas*	*syringae*	1420/1446 (98)	AY574914	5	AB697163
*Pseudomonas*	*reactans*	1240/1273 (97)	JN411452	5	AB695345
*Raoultella*	*ornithinolytica*	1360/1386 (98)	FJ823046	1	AB697156
*Rothia*	*dentocariosa*	1321/1330 (99)	CP002280	1	AB697164
*Rhodococcus*	*corynebacterioides*	1306/1313 (99)	AY167850	5	AB695346
*Sphingomonas*	*echinoides*	1335/1361 (98)	AB680957	5	AB695347
*Sphingomonas*	*yunnanensis*	1315/1330 (98)	EU730917	5	AB697165
*Stenotrophomonas*	*maltophilia*	1418/1432 (99)	AJ131117	1	AB697167

They are identified at the genus or species level on the basis of the 16S-rDNA sequence (*ca.* 1400 bp) for bacteria. “Identity” was shown by the number of nucleotide matched per number of nucleotide compared. “Frequency” indicates the numbers of detection out of 16 trials.

### Selection and specificity of oligonucleotide array for the detection of microbial species in the phyllosphere of apple trees

**Figure 1:**
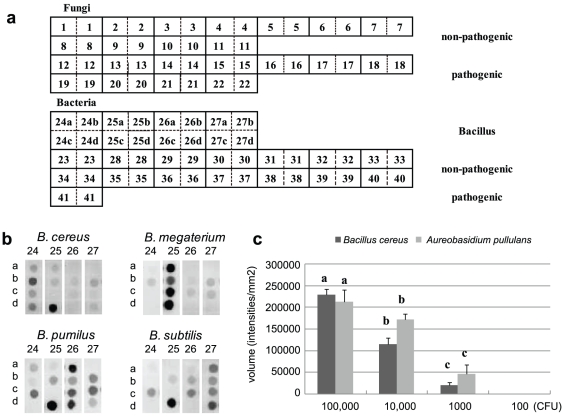
The arrangement, specificity, and quantitative nature of macroarray. (a) The arrangement of macroarray membrane. The numbers are corresponding to those in Table 3 and 4. Each array spots are duplicated except for those targeting four Bacillus species. (b) Four sets of four oligo-DNA arrays to discriminate four Bacillus species in the apple phyllosphere. (c) Quantitative analysis of the major fungus *A. pullulans* and bacterium *B. cereus* by macroarray. Error bars represent the standard deviation (±SD). Mean bars followed by different letters indicate significant differences by Tukey's test (P<0.05). Horizontal axis indicates the amounts (CFU) of *A. pullulans* and *B. cereus*. Vertical axis indicates volume measured by Quantity One.

On the basis of the results obtained by agar-plate culture method (see Table 1 and 2), we eliminated the species that were detected less than twice in the 16 trials, and selected 11 major non-pathogenic fungi and 18 non-pathogenic bacteria as the targets of macroarray analysis. In addition to these, 11 fungi pathogenic to apple trees and a fire blight pathogen, *Erwinia amylovora*, were added to the list of targets. Consequently, a total of 41 non-pathogenic and pathogenic fungi and bacteria were selected for macroarray analysis, and species-specific 40-bp oligo-DNA sequences were selected as array DNAs from the rDNA-ITS sequence of fungi and 16S-rDNA sequence of bacteria (Table 3 and 4). In case of the four *Bacillus* species selected, four each of 40-bp arrays were designed as described below (Figure 1; array No. 24–27).

**Table 3:** List of target fungi species for macroarray and the nucleotide sequences of oligo-DNA arrays.

	array No.	species	oligonucleotide array sequence
**non-pathogenic**			
	1	*Alternaria alternata*	ACCCTTGTCTTTTGCGTACTTCTTGTTTCCTTGGTGGGTT
	2	*Arthrinium sacchari*	AAGCTCGGTTGGAGGCACCTGCAGCTACCCTGTAGTTGCG
	3	*Aureobasidium pullulans*	AGAATTTATTCGAACGTCTGTCAAAGGAGAGGAACTCTGC
	4	*Botrytis byssoidea*	GAGTCTATGTCAGTAATGGCAGGCTCTAAAATCAGTGGCG
	5	*Cladosporium tenuissimum*	TCTAACCACCGGGATGTTCATAACCCTTTGTTGTCCGACT
	6	*Cystofilobasidium macerans*	CTCTCACCTCCAGCCTTCTTTAATTAGAGGTGTTGGGGCG
	7	*Epicoccum nigrum*	ATTACCTAGAGTTTGTGGACTTCGGTCTGCTACCTCTTAC
	8	*Fusarium equiseti*	TTTTTAGTGGAACTTCTGAGTAAAACAAACAAATAAATCA
	9	*Mucor racemosus*	GGATGACTGAGAGTCTCTTGATCGTCAGATCTCGAACCTC
	10	*Rhodotorula laryngis*	CACACATTTTAACACTATAGTATAAGAATGTAACAGTCTC
	11	*Cryptococcus victoriae*	TGAAACCTCACCCCACTTGGGTTTTTGCCTGAGCGGTGGT
**pathogenic**			
	12	*Alternaria mali*	AGCGCAGCACAAGTCGCACTCTCTATCAGCAAAGGTCTAG
	13	*Botrytis cinerea*	GTATTGAGTCTATGTCAGTAATGGCAGGCTCTAAAATCAG
	14	*Colletotrichum acutatum*	TTTACACGACGTCTCTTCTGAGTGGCACAAGCAAATAATT
	15	*Helicobasidium mompa*	TAGTCTAAGAATGTAAAGGACCCTTATAATTAATATAAAA
	16	*Monilinia fructicola*	CTATGTCAGTAATGGCAGGCTCTAAAATCAGTGGCGGCGC
	17	*Monilinia mali*	GTATTGAGCCCATGTCAGCGATGGCAGGCTCCAAAGTCAG
	18	*Penicillium expansum*	CCCGAACTCTGCCTGAAGATTGTCGTCTGAGTGAAAATAT
	19	*Schizophyllum commune*	CGGGCGGCGGTTGACTACGTCTACCTCACACCTTAAAGTA
	20	*Valsa ceratosperma*	CGCTGGCTGCCCCTCCCGCTCCGGGAGGGGGCCCGCCTCT
	21	*Venturia inaequalis*	ATTCGGCGCCTGGCGGGGACCACCCCCCGTTCGCGGGGGG
	22	*Diplocarpon mali*	CCTCGGGGCCGGCGGCTCCGGCTGCTGCGCCCTCGCCAGA

**Table 4:** List of target Bacteria species for macroarray and the nucleotide sequences of oligo-DNA arrays.

	array No.	species	oligonucleotide array sequence
**non-pathogenic**			
	23	*Acinetobacter johnsonii*	GTCGAGCGGGGAAGGGTAGCTTGCTACCTGACCTAGCGGC
	24a	*Bacillus cereus* or *B. thuringiensis*	TGGACCCGCGTCGCATTAGCTAGTTGGTGAGGTAACGGCT
	24b		GACTTTCTGGTCTGTAACTGACACTGAGGCGCGAAAGCGT
	24c		TAACTCCGGGAAACCGGGGCTAATACCGGATAACATTTTG
	24d		GGGGCTAATACCGGATAACATTTTGAACTGCATGGTTCGA
	25a	*B. megaterium*	GGCTTTTTGGTCTGTAACTGACGCTGAGGCGCGAAAGCGT
	25b		TGGGCCCGCGGTGCATTAGCTAGTTGGTGAGGTAACGGCT
	25c		TAACTTCGGGAAACCGAAGCTAATACCGGATAGGATCTTC
	25d		AGGATGAACGCTGGCGGCGTGCCTAATACATGCAAGTCGA
	26a	*B. pumilus*	TAACTCCGGGAAACCGGAGCTAATACCGGATAGTTCCTTG
	26b		TGGACCCGCGGCGCATTAACTAGTTGGTGAGGTAACGGCT
	26c		GACTCTCTGGTCTGTAACTGACGCTGAGGAGCGAAAGCGT
	26d		GGAGCTAATACCGGATAGTTCCTTGAACCGCATGGTTCAA
	27a	*B. subtilis*	GGGGCTAATACCGGATGGTTGTTTGAACCGCATGGTTCAA
	27b		TGGACCCGCGGCGCATTAGCTAGTTGGTGAGGTAACGGCT
	27c		GACTCTCTGGTCTGTAACTGACGCTGAGGAGCGAAAGCGT
	27d		TAACTCCGGGAAACCGGGGCTAATACCGGATGGTTGTTTG
	28	*Methylobacterium radiotolerans*	ACGCCCTTTTGGGGAAAGGTTTACTGCCGGAAGATCGGCC
	29	*Micrococcus luteus*	AACGATGAAGCCCAGCTTGCTGGGTGGATTAGTGGCGAAC
	30	*Paenibacillus amylolyticus*	AAGGAAACTGGAAAGACGGAGCAATCTGTCACTTGGGGAT
	31	*Paenibacillus polymyxa*	CCTGGTAGAGTAACTGCTCTTGAAGTGACGGTACCTGAGA
	32	*Pantoea agglomerans*	GGAAGGCGATGGGGTTAATAACCCTGTCGATTGACGTTAC
	33	*Pseudomonas graminis*	AGGAAGGGCAGTAAGCGAATACCTTGCTGTTTTGACGTTA
	34	*P. fluorescens*	GTTGGGAGGAAGGGCATTAACCTAATACGTTAGTGTTTTG
	35	*P. putida*	GGGCATTAACCTAATACGTTAGTGTTTTGACGTTACCGAC
	36	*P. syringae*	AGCGGCAGCACGGGTACTTGTACCTGGTGGCGAGCGGCGG
	37	*Rhodococcus corynebacterioides*	GAAAACCAGCAGCTCAACTGTTGGCTTGCAGGCGATACGG
	38	*Sphingomonas echinoides*	CTCAGGTTCGGAATAACAGCGAGAAATTGCTGCTAATACC
	39	*S. yunnanensis*	TCCAAAGATTTATCGCCAGAGGATGAGCCCGCGTGAGATT
	40	*Staphylococcus epidermidis*	AATATATTGAACCGCATGGTTCAATAGTGAAAGACGGTTT
**pathogenic**			
	41	*Erwinia amylovora*	GGGGAGGAAGGGTGAGAGGTTAATAACCTCCTGCATTGAC

Each array DNA was spotted (2 spots/array, except for *Bacillus* No. 24–27) onto a nylon membrane as shown in Figure 1a, *i*.*e.*, spot nos. 1–11 were arrays for non-pathogenic fungi; 12–22, for major fungal pathogens of apple trees; 23–40, for non-pathogenic bacteria; and 41, for *Erwinia amylovora*.

**Figure 2:**
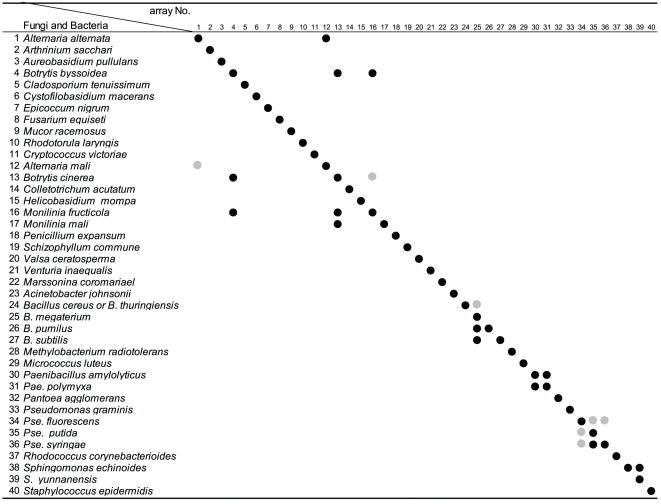
Schematic representation of specificity of oligo-DNA arrays. Arrays No. 1–40 are identical to those in Figure 1a. Black circles mean strong signals and gray ones mean weak non-specific cross-hybridization signals.

The specificity of the array was examined by hybridization with individual probes prepared separately from the 40 purified fungal and bacterial species. *E. amylovora*, however, could not be examined because the bacterium was unavailable in our laboratory. As summarized in Figure 2, most of the arrays with the exception of those listed below hybridized specifically with the corresponding species. Cross-hybridization was observed among those targeting *Botrytis elliptica* (or *byssoidea*), *B. cinerea*, *Monilinia fructicola* and *M. mali*, or *Alternaria alternate* and *A. mali*, or the four *Bacillus* spp.

In our preliminary examination, when we used Bacillus arrays No. 24a, 25a, 26a, and 27a, cross-hybridization was observed among the four *Bacillus* species (Fig. 2). Then we have designed supplementary combination of arrays to discriminate four Bacillus species. At present, it seemed difficult to distinguish these species by single DNA array, because the target 16S-rDNA sequences showed very high identity values. In order to distinguish the four *Bacillus* species, we have aligned all the four *Bacillus* 16S-rDNA sequences and selected three each of additional 40-nucleotide sequences unique to each species (Table 4; b, c, and d of array No. 24–27). The four sets of three oligo-DNAs, in addition to the original ones, were quantified, denatured, spotted, and hybridized with probes individually prepared from five *Bacillus* 16S-rDNAs. As the result, all the four arrays in each sets showed positive signals only in the homologous probe–array combinations (Fig. 1b). Consequently, the four *Bacillus* species can be distinguished using the four sets of four oligo-DNAs for each species.

### Simultaneous detection of major pathogenic and non-pathogenic fungi inhabiting the apple phyllosphere

**Figure 3:**
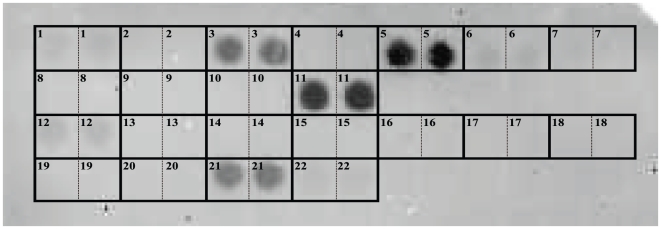
An image of macroarray hybridization for simultaneous detection of major pathogenic and non-pathogenic fungi in the phyllosphere of the apple trees. Arrangement of the arrays was the same to those in Figure 1a (Fungi). The arrays No. 3 (*A. pullulans*), 5 (*Cla. tenuissimum*), 11 (*Cry. victoriae*), and 21 (*V. inaequalis*) showed strong positive, and 6 (*Cys. macerans*) and 12 (*A. mali*) showed weak positive.

Simultaneous detection of major pathogenic and non-pathogenic fungi in the phyllosphere of the apple trees by the macroarray was examined with a probe prepared from apple leaves collected on August 27, 2009, from Orchard A-organic, where Alternaria blotch, scab, and Marssonina blotch were visibly epidemic. As a result, the macroarray allowed us to detect multiple signals ranging from strong to weak in the arrays not only for *Aureobasidium*, *Cladosporium*, *Cryptococcus*, and *Cystofilobasidium* genera of non-pathogenic fungi, but for *A. mali* (the pathogen causing Alternaria blotch), *Venturia inaequalis* (the pathogen causing apple scab), and *Diplocarpon mali* (the pathogen causing Marssonina blotch) of pathogenic fungi (Fig. 3). Notably, major pathogenic fungi such as *A. mali*, *V. inaequalis*, and *D. mali* were simultaneously detected, because these phytopathogenic fungi hardly be detected by agar-plate culture method, due to mainly by their inferior growth rate on the medium, even in the presence of severe disease symptoms on the leaves.

Consequently, the macroarray was able to simultaneously detect multiple species of major fungi, both pathogenic and non-pathogenic, inhabiting the apple phyllosphere. Various intensities of signals, ranging from strong to weak, supported that the data obtained are proportional to those inhabiting in the phyllosphere as we will examine in the next section.

### Quantitative nature of the macroarray

We have conducted two experiments to examine whether the signal intensity obtained by the macroarray is proportional to the actual microbial population in the phyllosphere; i.e., dilution kinetics of macroarray probe and direct nucleotide sequencing of microbial rDNAs in the phyllosphere.

First, in order to examine the dilution kinetics and the detection limit of the macroarray probe developed in this study, we selected *A. pullulans* from fungi and *B. cereus* from bacteria. *A. pullulans* is a ubiquitous yeast-like fungus predominating in apple phyllosphere. *B. cereus* is also a ubiquitous bacterium predominating in apple phylloshere. These fungus and bacterium suspension were prepared independently at the concentration of 10^5^ CFU/ml in distilled water and diluted serially from 10^1^ to 10^4^ by 10-fold dilution. According to the method described, we extracted DNA from 1 ml of each dilutions, prepared macroarray probes, and carried out macroarray hybridization. The macroarray analysis was repeated twice, and the relative ratios of microbes in the phyllosphere were estimated based on the average volume (intensities/mm^2^) of the four replicates.

As the result, the positive signals were obtained by the probes prepared from10^3^–10^5^ CFU in both of *A. pullulans* and *B. cereus*; indicating that the detection limit is 10^3^ CFU (Fig. 1c). The signal intensity was proportional to the microbial quantity ranging from 10^3^–10^5^ CFU.

**Figure 4:**
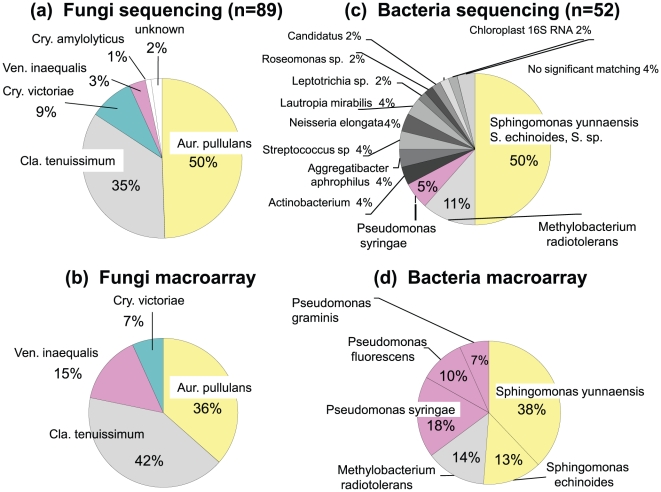
Comparison of nucleotide sequencing and macroarray for the detection of microbial rDNA population in the phyllosphere. Note that both of the fungal species and the ratio obtained by nucleotide sequencing (a) almost completely matched to the data obtained by macroarray (b). Although the minor bacteria species could not be detect by macroarray (d), but the major ones such as *Sphingomonas*, *Methylobacterium*, and *Pseudomonas* were consistent with both methods (c and d).

Next, by using the same field DNA preparation from the leaves corrected from the A-organic orchard in July 10th, 2010, we have conducted a comparative analysis of macroarray and direct nucleotide sequencing analyses of microbial rDNA and rDNA-ITS populations in the phyllosphere without culturing. To minimize the sampling bias as possible, the apple leaves collected from the above orchard (3 leaves from 1 position, 3 positions in 1 tree, and 3 trees in 1 orchard; namely, a total of 27 leaves per orchard), were crushed into small pieces in the liquid nitrogen, mixed well, and an aliquot (ca. 0.5-g) was used for the direct DNA extraction as described. The direct DNA extraction was repeated three times, PCR amplification was repeated three times for each DNA extracts, and finally nine PCR amplicons were combined for cloning and sequencing. After cloning the PCR amplicons, we have picked up 100 for fungal and 65 for bacterial independent clones, sequenced, and obtained 89 and 52 informative nucleotide sequences, respectively. These sequences were analyzed by BLAST and identified the species by the sequence similarity higher than 98%. As the result, fungi was consisted of 44 *A. pullulans* (50%; HQ909089), 31 *Cladosporium tenuissimum* (35%; JN689952, FQ832794), 8 *Cryptococcus victoriae* (9%; AF444645), 3 *Venturia inaequalis* (3%; EU035437), 1 *Cryptococcus aff. amylolyticus* (1%; EF363151), and 2 unknown species (2%; no significant similarity) (Fig. 4a). Bacteria was consisted of 26 *Sphingomonas* sp. (*S. yunnaensis*, *S. echinoides*, and sp.: 50%; AY336550, AY336556, AM989061, AB649018, AF395038, EU730917), 6 *Methylobacterium* radiotolerans and sp. (11%; AM989028, AF324201), 3 *Pseudomonas syringae* (5%; CP000075), 2 each of *Actinobacterium* sp. (4% AY275506, GU586309), *Aggregatibacter aphrophilus* (4%; EF605278), *Streptococcus* sp. (4%; AY518677, EU189961), *Neisseria elongate* (4%; L06171), *Lautropia mirabilis* (4%; GU397890), and etc. (AB594202, CP001277) (Fig. 4b).

Since the major species by the direct sequencing of apple leaf extract was completely matched to the data by the 3-year of culture method, it is unlikely that the apple leaves harbor unknown major unculturable species.

In the mean time, a macroarray probe was prepared from the same DNA preparation, and used for the macroarray analysis. The signal intensities of two each of dots per array were quantified by Quantity one. The macroarray analysis was repeated twice, and the relative ratios of microbes in the phyllosphere were estimated based on the average volume (intensities/mm^2^) of the four replicates. The result identified several non-pathogenic and pathogenic microbial inhabitants in the phyllosphere; i.e., the fungi of *A. pullulans* (with the relative ratio in the population of 36.5%), *C. tenuissimum* (41.7%), *V. inaequalis* (15.0%), and *Cystofilobasidium macerans* (6.7%) (Fig. 4c), and bacteria of *Sphingomonas yunnaensis* (38%), *P. syringae* (18%), *Methylobacterium radiotolerans* (14%), *Sphingomonas echinoides* (13%), *P. fluorescens* (10%), *P. graminis* (7%) (Fig. 4d).

These macroarray data were in agreement with those obtained by nucleotide sequencing, both in fungi and in bacteria, either in the predominant genera and their relative ratios in the population.

### Macroaray analysis of the seasonal changes in the major microbial inhabitants in the phyllosphere of the apple trees in the orchards under the different pest managements

The seasonal changes in the major fungi and bacteria inhabiting the phyllosphere of the apple trees in relation to diseases were examined by using leaf samples collected from the four apple orchards (A-chemical, A-organic, B-semi-chemical, and B-natural) from May 8 to October 29, 2009. All macroarray analyses were performed twice. It should be noted here that, unexpectedly but fortunately, the array “25d” targeting *B. megaterium* reacted stably and strongly to host chloroplast rDNA due to the high sequence homology, so that we used it as internal standard to normalize the signal intensity among the membranes.

**Figure 5:**
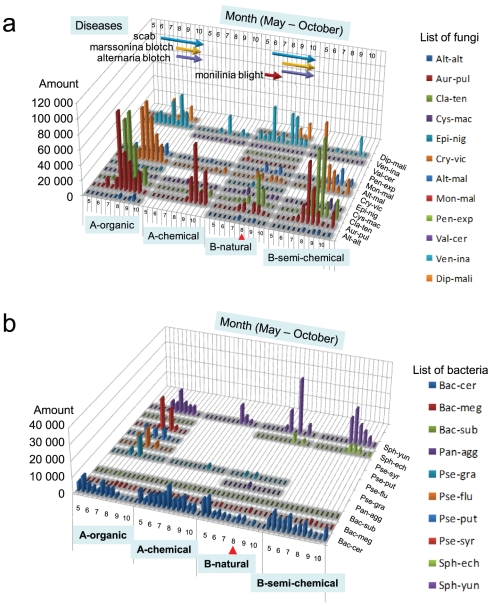
Seasonal changes in pathogenic and non-pathogenic fungi and bacteria inhabiting the apple phyllosphere in the four orchards. Histograms of seasonal changes of pathogenic and non-pathogenic fungi (a) and bacteria (b) detected from four orchards (A-chemical, A-organic, B-semi-chemical, B-natural) in 2009 May–October, by macroarray analysis. Y-axis shows relative amounts (average of two replicate) of each species quantified by QuantiOne software. The fungi and bacteria detected at least once in the orchard were indicated by grey background. The red vertical arrows indicate early-mid August when all the fungi and bacteria decreased to extremely lower levels. The major apple disease epidemics of Monilinia blight, scab, Marssonina blotch, and Alternaria blotch were indicated by horizontal arrows. Abbrebiations for fungi were *Alternaria alternata* (Alt-alt), *Aurerobasidum pullulans* (Aur-pil), *Cladosporium tenuissimum* (Cla-ten), *Cystofilobasidium macerans* (Cys-mac), *Epicoccum nigrum* (Epi-nig), *Cryptococcus victoriae* (Cry-vic), *Alternaria mali* (Alt-mal), *Monillinia mali* (Mon-mal), *Penicillium expansum* (Pen-exp), *Valsa ceratosperma* (Val-cer), *Venturia inaequalis* (Ven-ina), and *Diplocarpon mali* (Dip-mal), and for bacteria were *Bacillus cereus* (Bac-cer), *B. megaterium* (Bac-meg), *B. subtilis* (Bac-sub), *Pantoea aggromerans* (Pan-agg), *Pseudomons graminis* (Pse-gra), *P. fluorescens* (Pse-flu), *P. putida* (Pse-put), *P. syringae* (Pse-syr), *Sphingomonas echinoids* (Sph-ech), and *S. yunnanensis* (Sph-yun).

On the disease incidence in the four apple orchards, a conspicuous disease epidemic was not observed throughout the growing season in Orchards A-chemical or B-semi-chemical that were managed by normal cultivation with intensive spraying of chemical pesticides and chemical spraying reduced to less than half of the routine, respectively. Only scattered primary spots caused by secondary infections of Marssonina blotch (*D. mali*) were observed in Orchard B-semi-chemical in mid-October, albeit with minor damage. In Orchard A-organic managed by JAS organic, in contrast, the scab increased from June, and considerable amounts of Alternaria and Marssonina blotches were prevalent in August. In Orchard B-natural managed by natural farming, Monilinia blossom blight was the first epidemic in early May in the blooming period, and the scab started to develop from the end of May. Furthermore, Alternaria and Marssonina blotches started to develop from the end of June. Considerable amount of symptoms of apple scab, Alternaria blotch, and Marssonina blotch persisted at the end of the harvest season (Fig. 5a).

In addition, we analysed seasonal quantitative changes in the population of pathogenic and non-pathogenic fungi and bacteria inhabiting the phyllosphere of apple trees in the four orchards in 2009. First, the quantitative data obtained by fungal macroarray was summarized in Fig. 5a. In Orchard A-chemical, the amount of fungi was maintained at a low level from April to early June. Among the non-pathogenic fungi in the phyllosphere, *A. pullulans* was the most predominant species from September to October, and *C. tenuissimum* and *Cry. victoriae* were also detected in relatively high densities. A considerable amount of scab fungus *V. inaequalis* was first detected in late July; the fungus population decreased to a low level in early-to mid-August and increased again in late August, and was detected in October also.

In Orchard A-organic, at the same location as that in A-chemical, *A. pullulans* was again the most predominant species in the phyllosphere from late July to early October, and *C. tenuissimum* and *Cry. victoriae* were detected at levels similar to that of *A. pullulans*. A large amount of pathogenic fungi, such as *V. inaequalis*, *A. mali*, and *D. mali*, were detected from late May, late June, and early August, respectively, and continued to be detected until the end of October (the harvest season). It should be noted that the total amount of fungi in A-organic was 3 times that in A-chemical, which resulted from the high number of non-pathogenic fungi, such as *C. tenuissimum* and *Cry. victoriae*, in addition to the pathogenic *V. inaequalis*, *A. mali*, and *D. mali*, inhabiting the phyllosphere of trees in Orchard A-organic.

In Orchard B-semi-chemical, *A. pullulans*, *C. tenuissimum*, and *Cry. victoriae* predominated from late July to the end of the harvest season at levels almost equivalent to those observed in Orchard A-organic. Low levels of *A. mali* and *V. inaequalis* were detectable in early August and early October, respectively, indicating a potential merit of the reduced spraying of chemical fungicides in controlling the major fungal pathogens of apple trees, such as *V. inaequalis*, *A. mali*, and *D. mali*, without a serious negative impact on the major non-pathogenic fungi inhabiting the phyllosphere.

In Orchard B-natural, *V. inaequalis* was the most predominant species in the phyllosphere throughout the growing season, especially from late May to late October. *M. mali* was detectable in May, which is consistent with the observation that Monilinia blight was epidemic in May in the orchard. *A. mali* and *D. mali* were also detected from early August and late September to the end of growing season, respectively. It was noted that in this orchard, the numbers of both non-pathogenic and pathogenic fungi, with the exception of *V. inaequalis*, were suppressed to levels lower than those in the other orchards throughout the growing season. Although no chemical fungicide was sprayed, the numbers of *A. mali* and *D. mali* in Orchard B-natural were suppressed to levels lower than those in Orchard A-organic, indicating that disease control was more successful in B-natural. Meanwhile, 12 different species of pathogenic and non-pathogenic fungi were detected in this orchard, suggesting that the fungal diversity in the phyllosphere of the trees in Orchard B-natural was richer than that of the other orchards. In contrast, it was noted that the number of fungi inhabiting B-natural decreased to extremely low levels in mid-to late-August (Fig. 5a, red arrow).

Next, the quantitative data obtained by bacterial macroarray was summarized in Fig. 5b. Of all bacterial species, *Bacillus cereus* and *S. yunnaensis* predominated in all the orchards. *Pseudomonas* sp. were also detected in several samples. The variation in bacterial species was maximum in A-organic, *i.e., S. yunnaensis* from late July to late October, *P. fluorescens* from late July to the end of the growing season, and *P. syringae* from early August to the end of the growing season. *P. putida* and *B. subtilis* were also temporarily detected. In Orchard B-semi-chemical, like in Orchard A-organic, *Bacillus cereus*, *S. yunnaensis*, and a trace of *S. echinoids* were detected. In Orchard B-natural, like in Orchard B-semi-chemical, in addition to *Bacillus* and *Sphingomonas*, *Pantoea aggromerans* and *P. graminis* were also detected, meaning that bacterial diversity was a bit richer in Orchard B-natural than in Orchard B-semi-chemical. The bacterial biomass, except for *S. yunnaensis*, was apparently lower in this orchard than in the other orchards from June, and especially decreased in early August (Fig. 5b, red arrow).

In conclusion, *Bacillus*, *Pseudomonas*, and *Sphingomonas* genera predominated in all the orchards. The numbers of species detected in chemical fungicide-sprayed sites were apparently less than those detected in the organic sites.

## Discussion

In a 3-year study (2006–2008) using agar-plate culture method, we detected 32 fungal and 34 bacterial species inhabiting the phyllosphere of apple trees in northern Japan. Because we used PDA and King's B agar for isolating fungi and bacteria, respectively, most of the isolates were non-pathogenic saprophytes with higher growth rates in these media. In contrast, major fungal pathogens of apple trees, including the agents causing scab, Alternaria blotch, and Marssonina blotch, were not detected in these experiments, despite the presence of severe symptoms. *Aureobasidium*, *Cladosporium*, *Alternaria*, *Rhodotorula*, and *Cystofilobasidium* genera were the predominant fungal species that showed extensive growth. This is consistent with the findings of previous studies conducted in New Zealand and Switzerland that showed that *A. arborescens*, *A. pullulans*, *C. tenuissimum*, and *A. mali* were frequently isolated from apple leaves [8], [21]. *Bacillus*, *Pseudomonas*, and *Sphingomonas* genera were the predominant bacterial species that showed extensive growth.

On the basis of our results, we selected 41 species of major pathogenic and non-pathogenic fungi and bacteria inhabiting the phyllosphere of apple trees in northern Japan. In the preliminary steps, we examined nearly full-length rDNA-ITS regions for cDNA arrays, but they lacked specificity (data not shown). We also examined 30-bp oligo-DNAs for arrays, but their sensitivities were not high enough (data not shown). Finally, we adapted 40-bp oligo-DNAs specific for each fungal rDNA-ITS region or bacterial 16S-rDNA and established an oligo-DNA macroarray for analyzing/monitoring richness of the major microbial species in the phyllosphere of apple trees. Most of the arrays specifically identified the target species. However, in some cases, *Alternaria* and related fungal species or *Bacillus* spp. could not be clearly distinguished because of cross-hybridization.

Sholberg et al. [22] used 19- to 25-bp-long oligo-DNA from the ribosomal spacer regions of bacterial and fungal pathogens to identify and monitor economically important apple diseases. The DNA array correctly identified *B. cinerea*, *Penicillium expansum*, *Podosphaera leucotricha*, *V. inaequalis*, and *E. amylovora*, and eliminated closely related species. When the array was used to monitor *V. inaequalis* ascospores collected from spore traps located in orchards, it confirmed the presence of ascospores as predicted by the disease-forecasting model, suggesting that the DNA array can be a useful tool for epidemiological studies. By using the macroarray developed, we have successfully detected pathogens such as *A. mali*, *Valsa ceratosperma*, and *V. inaequalis* even from the orchards where the disease symptoms were virtually invisible, indicating that the macroarrray is actually useful for monitoring economically important apple diseases.

Zhang et al. [23] developed macroarray for the detection of solanaceous plant pathogens in the *Fusarium solani* species complex. Thirty-three 17- to 27-bp-long oligonucleotides were designed from the rDNA-ITS sequences of 17 isolates, which belonged to 12 phylogenetically related species. The array was validated by testing inoculated greenhouse samples and diseased field plant samples. Furthermore, Zhang et al. [24] designed 105 17- to 27-bp-long oligonucleotides specific for 25 pathogens of solanaceous crops on the basis of the rRNA-ITS gene sequence. They adapted at least 2 specific oligonucleotides per pathogen to distinguish between closely related species. Although both of the research purpose and target species were totally different, we specifically detected most of 41 major pathogenic and non-pathogenic fungi and bacteria using single array per species by taking the data obtained by preliminary surveillance in consideration. The strategy we employed here is definitely useful for monitoring the richness in the major microbial diversity in a specific host or restricted ecological environment.

The macroarray was adapted to analyze seasonal changes in major epiphytic microbial populations in the phyllosphere of apple trees in the growing season of 2009 in the four representative orchards in northern Japan. The findings were consistent with the data obtained in the study conducted during 2006–2008 seasons with an agar-plate culture method and with our field observations on the disease epidemics in these orchards. For example, the signal intensities of the arrays for *Aureobasidium*, *Cladosporium*, and *Cryptococcus* fungal genera and *Bacillus*, *Pseudomonas*, and *Sphingomonas* bacterial genera were clearly visible and changed strong to week throughout the growing season in most of the orchards. It should be noted that similar results were independently obtained by employing culture methods in New Zealand and Switzerland [8], [21], suggesting that the predominant species in the phyllosphere of apple trees maybe the same all around the world. Furthermore, as a merit, not only the major saprophytic epiphytes, but also major pathogenic fungi, such as *A. mali*, *V. inaequalis*, and *D. mali*, which were not detected by agar-plate culture method, were certainly detected by this macroarray technique. These phytopathogenic fungi were also detectable in the chemical fungicide-sprayed orchards (A-chemical and B-semi-chemical) in the absence of foliar symptoms, suggesting that the macroarray is sensitive enough to monitor changes in the richness of phytopathogenic and non-phytopathogenic fungi and bacteria inhabiting the phyllosphere of apple trees.

Several interesting differences could be seen in the epiphytic microbial diversity in the phyllosphere of apple trees in the orchards with or without employing intensive spraying of chemical fungicides. Firstly, the intensive spraying of chemical fungicides (Orchard A-chemical) reduced the diversity and abundance of both fungi and bacteria in the phyllosphere of apple trees. This is partly consistent with the former observation with Golden Delicious apples in Spain that the fungicide regime on apple trees significantly decreased the total filamentous fungal population; however, bacterial populations were higher on the apples from fungicide-treated plots [25]. Secondary, in the Orchard B-semi-chemical, the development of diseases was successfully controlled without giving adverse impact on the epiphytic microbial diversity in the phyllosphere of apple trees. Finally, a couple of unexpected and interesting findings were obtained in Orchard B-natural, the “natural-farming” orchard; *i.e.*, the abundance of fungal species was the highest in this orchard but the amounts of individual species, with the exception of phytopathogenic *V. inaequalis*, were apparently lower than the others throughout the growing season as represented by *A. pullulans*. Because the orchard has been maintained to produce apples without relying on any chemical fungicides, it is essential to conduct a more intensive and advanced analysis in relation to microbial diversity and disease control.

The macroarray technique presented here is a strong tool to monitor the complexities of microbial species or the community structures of microbial flora in the phyllosphere of apple trees and identify key species antagonistic, supportive or co-operative to specific pathogens in the orchard managed under different environmental conditions.
